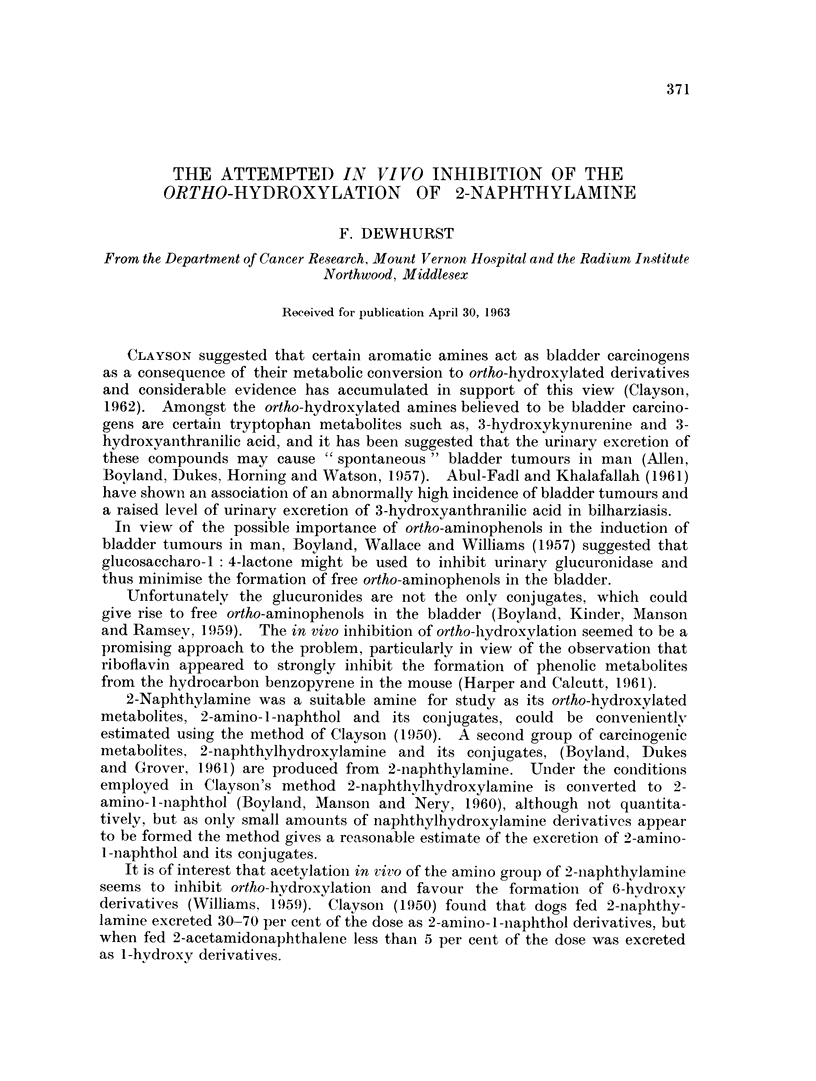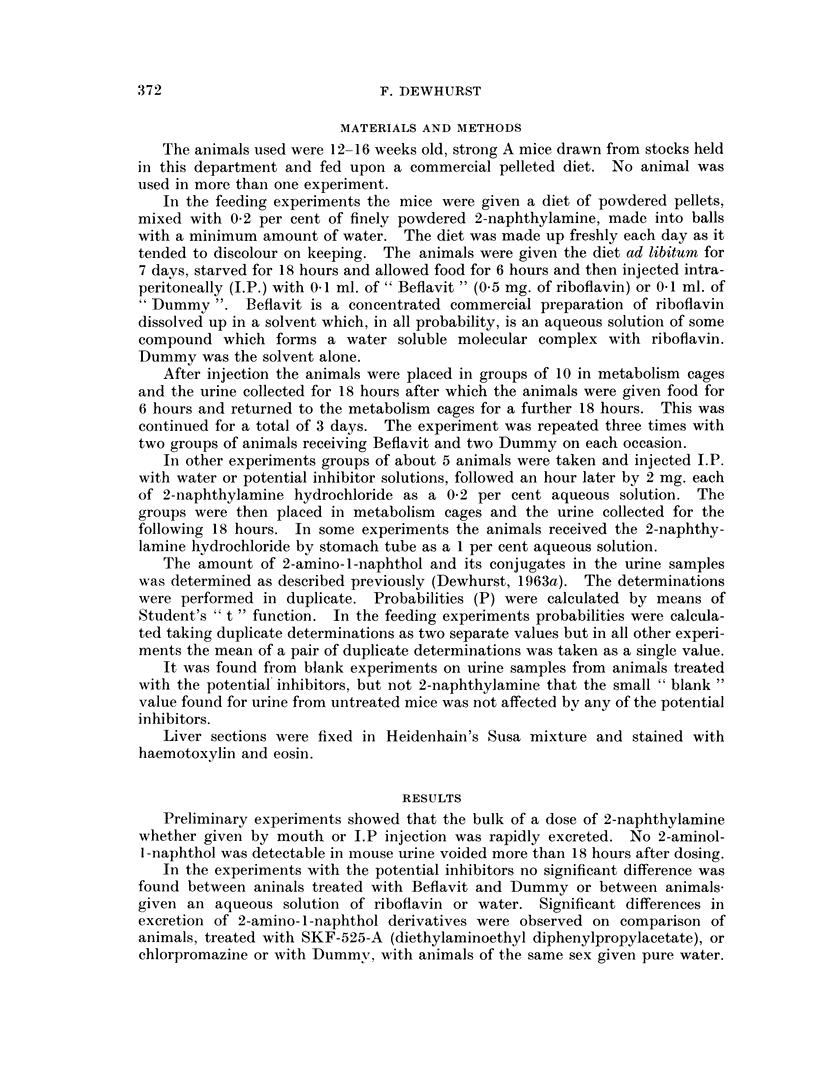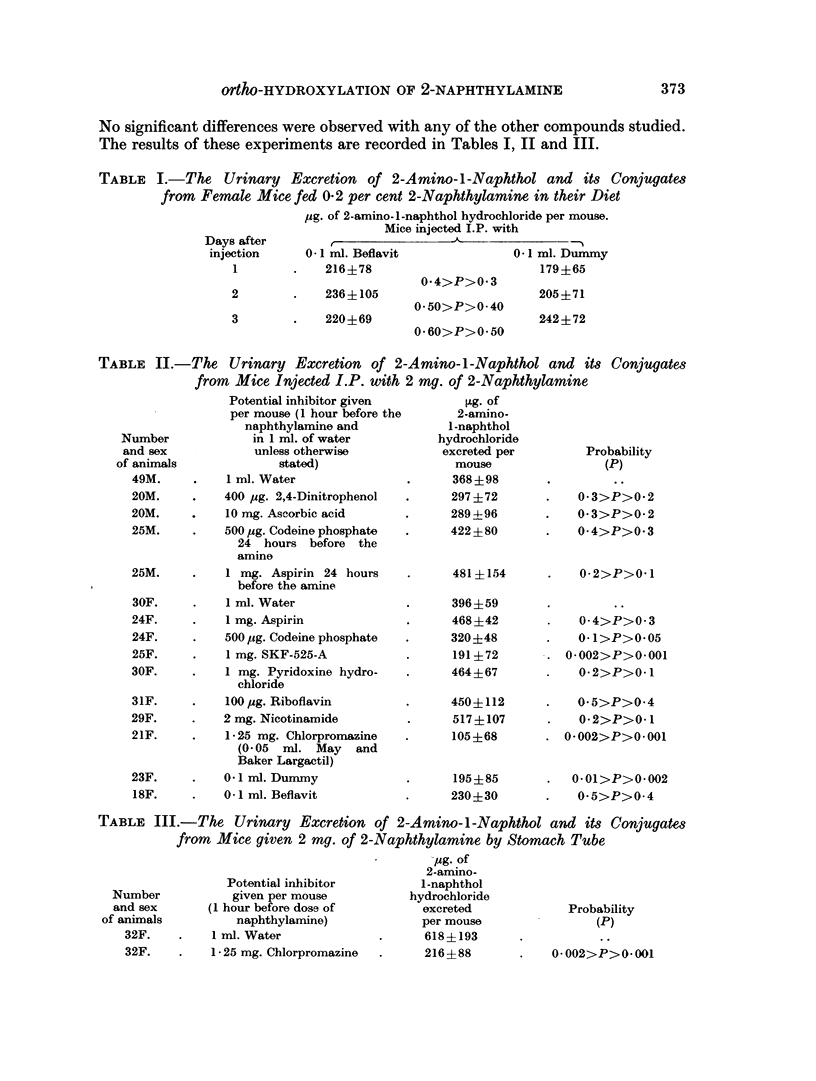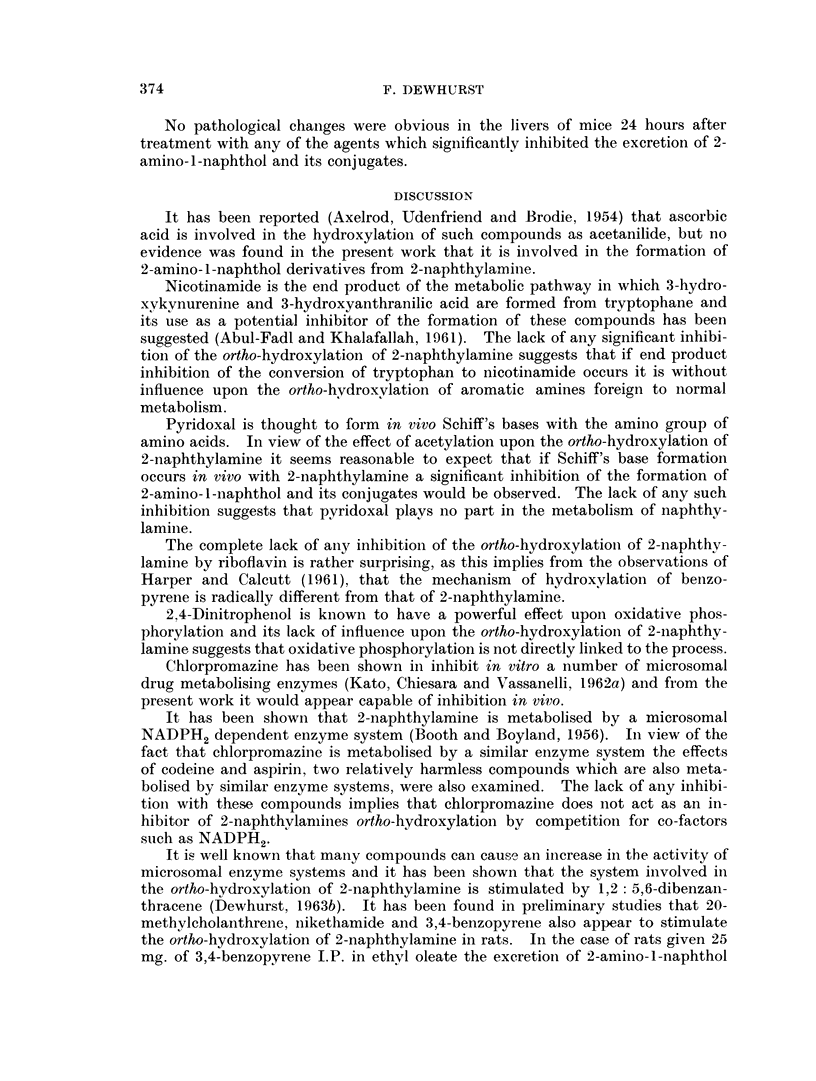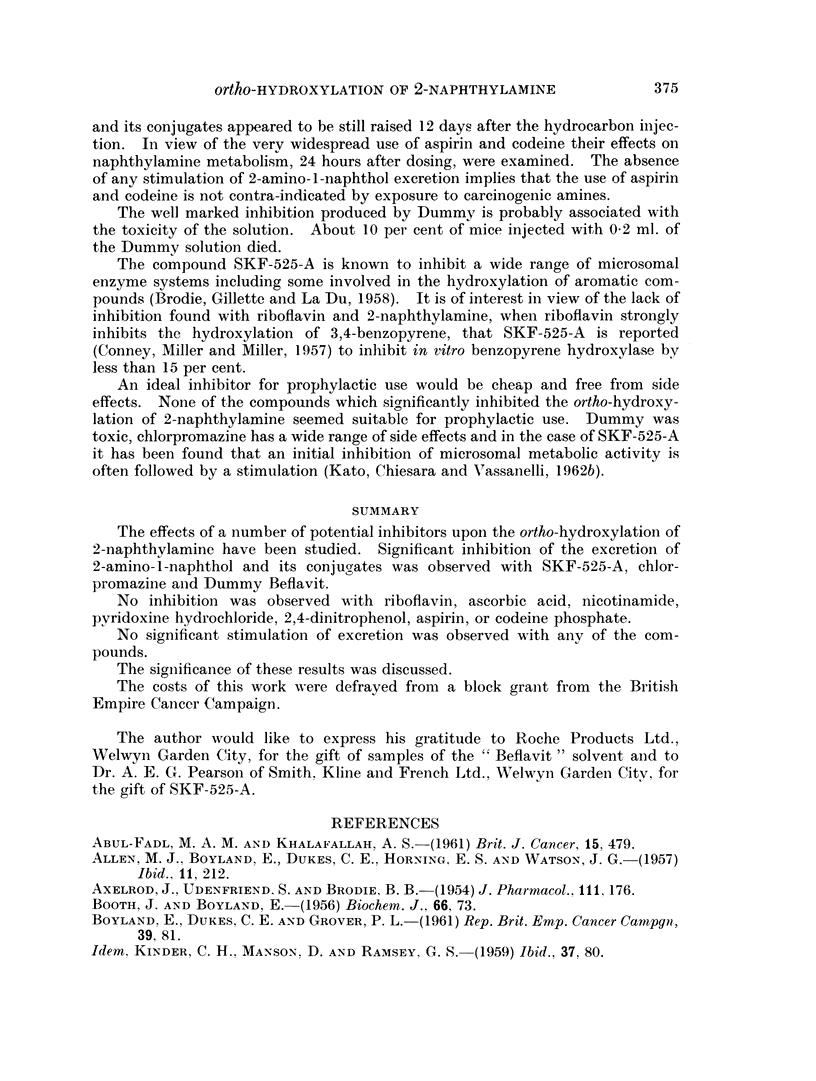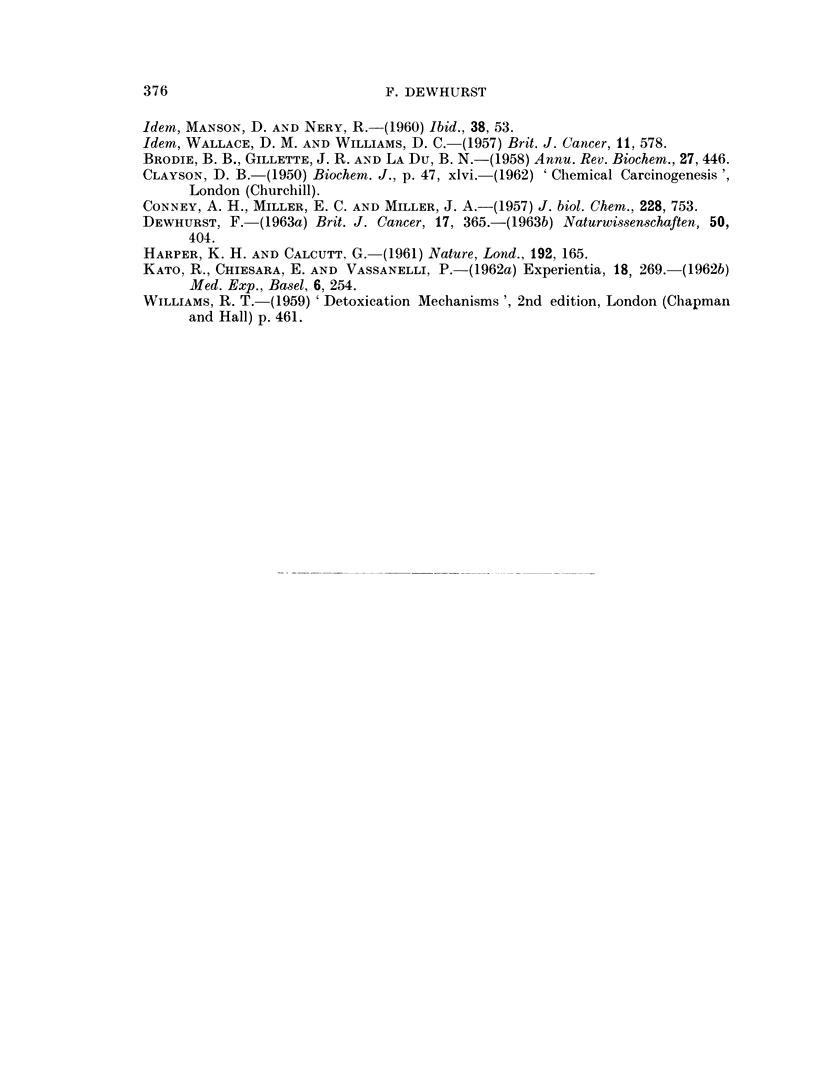# The Attempted In Vivo Inhibition of the Ortho-hydroxylation of 2-Naphthylamine

**DOI:** 10.1038/bjc.1963.51

**Published:** 1963-06

**Authors:** F. Dewhurst


### THE ATTEMPTED *IN VIVO* INHIBITION OF THE *ORTHO*-HYDROXYLATION OF 2-NAPHTHYLAMINE

F. DEWHURST

*From the Department of Cancer Research, Mount Vernon Hospital and the Radium Institute Northwood, Middlesex*

Received for publication April 30, 1963

CLAYSON suggested that certain aromatic amines act as bladder carcinogens as a consequence of their metabolic conversion to *ortho*-hydroxylated derivatives and considerable evidence has accumulated in support of this view (Clayson, 1962). Amongst the *ortho*-hydroxylated amines believed to be bladder carcinogens are certain tryptophan metabolites such as, 3-hydroxykynurenine and 3-hydroxyanthranilic acid, and it has been suggested that the urinary excretion of these compounds may cause " spontaneous " bladder tumours in man (Allen, Boyland, Dukes, Horning and Watson, 1957). Abul-Fadl and Khalafallah (1961) have shown an association of an abnormally high incidence of bladder tumours and a raised level of urinary excretion of 3-hydroxyanthranilic acid in bilharziasis.

In view of the possible importance of *ortho*-aminophenols in the induction of bladder tumours in man, Boyland, Wallace and Williams (1957) suggested that glucosaccharo-1 : 4-lactone might be used to inhibit urinary glucuronidase and thus minimise the formation of free *ortho*-aminophenols in the bladder.

Unfortunately the glucuronides are not the only conjugates, which could give rise to free *ortho*-aminophenols in the bladder (Boyland, Kinder, Manson and Ramsey, 1959). The *in vivo* inhibition of *ortho*-hydroxylation seemed to be a promising approach to the problem, particularly in view of the observation that riboflavin appeared to strongly inhibit the formation of phenolic metabolites from the hydrocarbon benzopyrene in the mouse (Harper and Calcutt, 1961).

2-Naphthylamine was a suitable amine for study as its *ortho*-hydroxylated metabolites, 2-amino-1-naphthol and its conjugates, could be conveniently estimated using the method of Clayson (1950). A second group of carcinogenic metabolites, 2-naphthylhydroxylamine and its conjugates, (Boyland, Dukes and Grover, 1961) are produced from 2-naphthylamine. Under the conditions employed in Clayson's method 2-naphthylhydroxylamine is converted to 2-amino-1-naphthol (Boyland, Manson and Nery, 1960), although not quantitatively, but as only small amounts of naphthylhydroxylamine derivatives appear to be formed the method gives a reasonable estimate of the excretion of 2-amino-1-naphthol and its conjugates.

It is of interest that acetylation *in vivo* of the amino group of 2-naphthylamine seems to inhibit *ortho*-hydroxylation and favour the formation of 6-hydroxy derivatives (Williams, 1959). Clayson (1950) found that dogs fed 2-naphthylamine excreted 30–70 per cent of the dose as 2-amino-1-naphthol derivatives, but when fed 2-acetamidonaphthalene less than 5 per cent of the dose was excreted as 1-hydroxy derivatives.

### MATERIALS AND METHODS

The animals used were 12–16 weeks old, strong A mice drawn from stocks held in this department and fed upon a commercial pelleted diet. No animal was used in more than one experiment.

In the feeding experiments the mice were given a diet of powdered pellets, mixed with 0·2 per cent of finely powdered 2-naphthylamine, made into balls with a minimum amount of water. The diet was made up freshly each day as it tended to discolour on keeping. The animals were given the diet *ad libitum* for 7 days, starved for 18 hours and allowed food for 6 hours and then injected intraperitoneally (I.P.) with 0·1 ml. of " Beflavit " (0·5 mg. of riboflavin) or 0·1 ml. of " Dummy ". Beflavit is a concentrated commercial preparation of riboflavin dissolved up in a solvent which, in all probability, is an aqueous solution of some compound which forms a water soluble molecular complex with riboflavin. Dummy was the solvent alone.

After injection the animals were placed in groups of 10 in metabolism cages and the urine collected for 18 hours after which the animals were given food for 6 hours and returned to the metabolism cages for a further 18 hours. This was continued for a total of 3 days. The experiment was repeated three times with two groups of animals receiving Beflavit and two Dummy on each occasion.

In other experiments groups of about 5 animals were taken and injected I.P. with water or potential inhibitor solutions, followed an hour later by 2 mg. each of 2-naphthylamine hydrochloride as a 0·2 per cent aqueous solution. The groups were then placed in metabolism cages and the urine collected for the following 18 hours. In some experiments the animals received the 2-naphthylamine hydrochloride by stomach tube as a 1 per cent aqueous solution.

The amount of 2-amino-1-naphthol and its conjugates in the urine samples was determined as described previously (Dewhurst, 1963*a*). The determinations were performed in duplicate. Probabilities (P) were calculated by means of Student's " t " function. In the feeding experiments probabilities were calculated taking duplicate determinations as two separate values but in all other experiments the mean of a pair of duplicate determinations was taken as a single value.

It was found from blank experiments on urine samples from animals treated with the potential inhibitors, but not 2-naphthylamine that the small " blank " value found for urine from untreated mice was not affected by any of the potential inhibitors.

Liver sections were fixed in Heidenhain's Susa mixture and stained with haemotoxylin and eosin.

### RESULTS

Preliminary experiments showed that the bulk of a dose of 2-naphthylamine whether given by mouth or I.P injection was rapidly excreted. No 2-aminol-1-naphthol was detectable in mouse urine voided more than 18 hours after dosing.

In the experiments with the potential inhibitors no significant difference was found between aninals treated with Beflavit and Dummy or between animals· given an aqueous solution of riboflavin or water. Significant differences in excretion of 2-amino-1-naphthol derivatives were observed on comparison of animals, treated with SKF-525-A (diethylaminoethyl diphenylpropylacetate), or chlorpromazine or with Dummy, with animals of the same sex given pure water.

No significant differences were observed with any of the other compounds studied. The results of these experiments are recorded in Tables I, II and III.

TABLE I.—*The Urinary Excretion of 2-Amino-1-Naphthol and its Conjugates from Female Mice fed 0·2 per cent 2-Naphthylamine in their Diet*

| Days after injection | μg. of 2-amino-1-naphthol hydrochloride per mouse. Mice injected I.P. with: 0·1 ml. Beflavit | | 0·1 ml. Dummy |
|---|---|---|---|
| 1 | 216±78 | | 179±65 |
| | | 0·4>P>0·3 | |
| 2 | 236±105 | | 205±71 |
| | | 0·50>P>0·40 | |
| 3 | 220±69 | | 242±72 |
| | | 0·60>P>0·50 | |

TABLE II.—*The Urinary Excretion of 2-Amino-1-Naphthol and its Conjugates from Mice Injected I.P. with 2 mg. of 2-Naphthylamine*

| Number and sex of animals | Potential inhibitor given per mouse (1 hour before the naphthylamine and in 1 ml. of water unless otherwise stated) | μg. of 2-amino-1-naphthol hydrochloride excreted per mouse | Probability (P) |
|---|---|---|---|
| 49M. | 1 ml. Water | 368±98 | .. |
| 20M. | 400 μg. 2,4-Dinitrophenol | 297±72 | 0·3>P>0·2 |
| 20M. | 10 mg. Ascorbic acid | 289±96 | 0·3>P>0·2 |
| 25M. | 500 μg. Codeine phosphate 24 hours before the amine | 422±80 | 0·4>P>0·3 |
| 25M. | 1 mg. Aspirin 24 hours before the amine | 481±154 | 0·2>P>0·1 |
| 30F. | 1 ml. Water | 396±59 | .. |
| 24F. | 1 mg. Aspirin | 468±42 | 0·4>P>0·3 |
| 24F. | 500 μg. Codeine phosphate | 320±48 | 0·1>P>0·05 |
| 25F. | 1 mg. SKF-525-A | 191±72 | 0·002>P>0·001 |
| 30F. | 1 mg. Pyridoxine hydrochloride | 464±67 | 0·2>P>0·1 |
| 31F. | 100 μg. Riboflavin | 450±112 | 0·5>P>0·4 |
| 29F. | 2 mg. Nicotinamide | 517±107 | 0·2>P>0·1 |
| 21F. | 1·25 mg. Chlorpromazine (0·05 ml. May and Baker Largactil) | 105±68 | 0·002>P>0·001 |
| 23F. | 0·1 ml. Dummy | 195±85 | 0·01>P>0·002 |
| 18F. | 0·1 ml. Beflavit | 230±30 | 0·5>P>0·4 |

TABLE III.—*The Urinary Excretion of 2-Amino-1-Naphthol and its Conjugates from Mice given 2 mg. of 2-Naphthylamine by Stomach Tube*

| Number and sex of animals | Potential inhibitor given per mouse (1 hour before dose of naphthylamine) | μg. of 2-amino-1-naphthol hydrochloride excreted per mouse | Probability (P) |
|---|---|---|---|
| 32F. | 1 ml. Water | 618±193 | .. |
| 32F. | 1·25 mg. Chlorpromazine | 216±88 | 0·002>P>0·001 |

No pathological changes were obvious in the livers of mice 24 hours after treatment with any of the agents which significantly inhibited the excretion of 2-amino-1-naphthol and its conjugates.

### DISCUSSION

It has been reported (Axelrod, Udenfriend and Brodie, 1954) that ascorbic acid is involved in the hydroxylation of such compounds as acetanilide, but no evidence was found in the present work that it is involved in the formation of 2-amino-1-naphthol derivatives from 2-naphthylamine.

Nicotinamide is the end product of the metabolic pathway in which 3-hydroxykynurenine and 3-hydroxyanthranilic acid are formed from tryptophane and its use as a potential inhibitor of the formation of these compounds has been suggested (Abul-Fadl and Khalafallah, 1961). The lack of any significant inhibition of the *ortho*-hydroxylation of 2-naphthylamine suggests that if end product inhibition of the conversion of tryptophan to nicotinamide occurs it is without influence upon the *ortho*-hydroxylation of aromatic amines foreign to normal metabolism.

Pyridoxal is thought to form *in vivo* Schiff's bases with the amino group of amino acids. In view of the effect of acetylation upon the *ortho*-hydroxylation of 2-naphthylamine it seems reasonable to expect that if Schiff's base formation occurs *in vivo* with 2-naphthylamine a significant inhibition of the formation of 2-amino-1-naphthol and its conjugates would be observed. The lack of any such inhibition suggests that pyridoxal plays no part in the metabolism of naphthylamine.

The complete lack of any inhibition of the *ortho*-hydroxylation of 2-naphthylamine by riboflavin is rather surprising, as this implies from the observations of Harper and Calcutt (1961), that the mechanism of hydroxylation of benzopyrene is radically different from that of 2-naphthylamine.

2,4-Dinitrophenol is known to have a powerful effect upon oxidative phosphorylation and its lack of influence upon the *ortho*-hydroxylation of 2-naphthylamine suggests that oxidative phosphorylation is not directly linked to the process.

Chlorpromazine has been shown in inhibit *in vitro* a number of microsomal drug metabolising enzymes (Kato, Chiesara and Vassanelli, 1962*a*) and from the present work it would appear capable of inhibition *in vivo*.

It has been shown that 2-naphthylamine is metabolised by a microsomal $NADPH_2$ dependent enzyme system (Booth and Boyland, 1956). In view of the fact that chlorpromazine is metabolised by a similar enzyme system the effects of codeine and aspirin, two relatively harmless compounds which are also metabolised by similar enzyme systems, were also examined. The lack of any inhibition with these compounds implies that chlorpromazine does not act as an inhibitor of 2-naphthylamines *ortho*-hydroxylation by competition for co-factors such as $NADPH_2$.

It is well known that many compounds can cause an increase in the activity of microsomal enzyme systems and it has been shown that the system involved in the *ortho*-hydroxylation of 2-naphthylamine is stimulated by 1,2 : 5,6-dibenzanthracene (Dewhurst, 1963*b*). It has been found in preliminary studies that 20-methylcholanthrene, nikethamide and 3,4-benzopyrene also appear to stimulate the *ortho*-hydroxylation of 2-naphthylamine in rats. In the case of rats given 25 mg. of 3,4-benzopyrene I.P. in ethyl oleate the excretion of 2-amino-1-naphthol and its conjugates appeared to be still raised 12 days after the hydrocarbon injection. In view of the very widespread use of aspirin and codeine their effects on naphthylamine metabolism, 24 hours after dosing, were examined. The absence of any stimulation of 2-amino-1-naphthol excretion implies that the use of aspirin and codeine is not contra-indicated by exposure to carcinogenic amines.

The well marked inhibition produced by Dummy is probably associated with the toxicity of the solution. About 10 per cent of mice injected with 0·2 ml. of the Dummy solution died.

The compound SKF-525-A is known to inhibit a wide range of microsomal enzyme systems including some involved in the hydroxylation of aromatic compounds (Brodie, Gillette and La Du, 1958). It is of interest in view of the lack of inhibition found with riboflavin and 2-naphthylamine, when riboflavin strongly inhibits the hydroxylation of 3,4-benzopyrene, that SKF-525-A is reported (Conney, Miller and Miller, 1957) to inhibit *in vitro* benzopyrene hydroxylase by less than 15 per cent.

An ideal inhibitor for prophylactic use would be cheap and free from side effects. None of the compounds which significantly inhibited the *ortho*-hydroxylation of 2-naphthylamine seemed suitable for prophylactic use. Dummy was toxic, chlorpromazine has a wide range of side effects and in the case of SKF-525-A it has been found that an initial inhibition of microsomal metabolic activity is often followed by a stimulation (Kato, Chiesara and Vassanelli, 1962*b*).

### SUMMARY

The effects of a number of potential inhibitors upon the *ortho*-hydroxylation of 2-naphthylamine have been studied. Significant inhibition of the excretion of 2-amino-1-naphthol and its conjugates was observed with SKF-525-A, chlorpromazine and Dummy Beflavit.

No inhibition was observed with riboflavin, ascorbic acid, nicotinamide, pyridoxine hydrochloride, 2,4-dinitrophenol, aspirin, or codeine phosphate.

No significant stimulation of excretion was observed with any of the compounds.

The significance of these results was discussed.

The costs of this work were defrayed from a block grant from the British Empire Cancer Campaign.

The author would like to express his gratitude to Roche Products Ltd., Welwyn Garden City, for the gift of samples of the " Beflavit " solvent and to Dr. A. E. G. Pearson of Smith, Kline and French Ltd., Welwyn Garden City, for the gift of SKF-525-A.

### REFERENCES

ABUL-FADL, M. A. M. AND KHALAFALLAH, A. S.—(1961) *Brit. J. Cancer*, **15**, 479.
ALLEN, M. J., BOYLAND, E., DUKES, C. E., HORNING, E. S. AND WATSON, J. G.—(1957) *Ibid.*, **11**, 212.
AXELROD, J., UDENFRIEND, S. AND BRODIE, B. B.—(1954) *J. Pharmacol.*, **111**, 176.
BOOTH, J. AND BOYLAND, E.—(1956) *Biochem. J.*, **66**, 73.
BOYLAND, E., DUKES, C. E. AND GROVER, P. L.—(1961) *Rep. Brit. Emp. Cancer Campgn*, **39**, 81.
*Idem*, KINDER, C. H., MANSON, D. AND RAMSEY, G. S.—(1959) *Ibid.*, **37**, 80.

*Idem*, MANSON, D. AND NERY, R.—(1960) *Ibid.*, **38**, 53.
*Idem*, WALLACE, D. M. AND WILLIAMS, D. C.—(1957) *Brit. J. Cancer*, **11**, 578.
BRODIE, B. B., GILLETTE, J. R. AND LA DU, B. N.—(1958) *Annu. Rev. Biochem.*, **27**, 446.
CLAYSON, D. B.—(1950) *Biochem. J.*, p. 47, xlvi.—(1962) ' Chemical Carcinogenesis ', London (Churchill).
CONNEY, A. H., MILLER, E. C. AND MILLER, J. A.—(1957) *J. biol. Chem.*, **228**, 753.
DEWHURST, F.—(1963*a*) *Brit. J. Cancer*, **17**, 365.—(1963*b*) *Naturwissenschaften*, **50**, 404.
HARPER, K. H. AND CALCUTT, G.—(1961) *Nature, Lond.*, **192**, 165.
KATO, R., CHIESARA, E. AND VASSANELLI, P.—(1962*a*) Experientia, **18**, 269.—(1962*b*) *Med. Exp., Basel*, **6**, 254.
WILLIAMS, R. T.—(1959) ' Detoxication Mechanisms ', 2nd edition, London (Chapman and Hall) p. 461.